# Adherence to antihypertensive treatment during the COVID-19 pandemic: findings from a cross-sectional study

**DOI:** 10.1186/s40885-022-00219-0

**Published:** 2022-12-01

**Authors:** Mayra Cristina da Luz Pádua Guimarães, Juliana Chaves Coelho, Juliano dos Santos, Camila Braga de Oliveira Higa, Carime Farah Flórido, Renata Jae Won Lee, Grazielli Soares Paes, Giovanio Vieira da Silva, Luciano Ferreira Drager, Angela Maria Geraldo Pierin

**Affiliations:** 1 University of São Paulo Nursing School, São Paulo, Brazil; 2 Beneficência Portuguesa Hospital in São Paulo, São Paulo, Brazil; 3 Cancer Hospital III, National Cancer Institute of Brazil, Rio de Janeiro, Brazil; 4 University of São Paulo Medical School, São Paulo, Brazil

**Keywords:** Hypertension, Adherence, COVID-19, Drug treatment

## Abstract

**Background:**

Nonadherence to antihypertensive treatment is one of the main causes of the lack of blood pressure (BP) control. The coronavirus disease (COVID-19) pandemic imposes substantial social restriction impairing the medical care routine, which may influence adherence to the antihypertensive treatment. To assess the rate of nonadherence to antihypertensive drug treatment during the COVID-19 pandemic.

**Methods:**

This is a cross-sectional study evaluating hypertensive adult patients from a tertiary outpatient clinic. From March to August 2020, patients were interviewed by telephone during the social distancing period of the COVID-19 pandemic. We evaluated biosocial data, habits, attitudes, and treatment adherence using the 4-item Morisky Green Levine Scale during the social distancing. Uncontrolled BP was defined by BP ≥ 140/90 mmHg. Clinical and prescription variables for drug treatment were obtained from the electronic medical record. We performed a multivariate analysis to determine the predictors of nonadherence to BP treatment.

**Results:**

We studied 281 patients (age 66 ± 14 years, 60.5% white, 62.3% women, mean education of 9.0 ± 4 years of study). We found that 41.3% of the individuals reported poor adherence to antihypertensive drug treatment and 48.4% had uncontrolled BP. Subsample data identified that adherence was worse during the pandemic than in the previous period. The variables that were independently associated with the nonadherence during the pandemic period were black skin color (odds ratio [OR], 2.62; 95% confidence interval [CI], 1.46–4.68), and intermittent lack of access to antihypertensive medication during the pandemic (OR, 2.56; 95% CI, 1.11–5.89).

**Conclusions:**

Beyond traditional variables associated with poor adherence, the lack of availability of antihypertensive medications during the study underscore the potential role of pandemic on hypertension burden.

## Background

The pandemic caused by the coronavirus, severe acute respiratory syndrome coronavirus 2, namely coronavirus disease (COVID-19), has been challenging to the global health system [1–4]. According to the World Health Organization, to date, more than 520 million people have been infected and more than six million have died. In Brazil, the panorama is no different. Estimates show that the number of deaths has already exceeded 664,000 and more than 30 million people have been infected [5].

Since the beginning of the pandemic, several factors have been associated with poor prognosis [6–8]. Review studies show that hypertension (56.6%) and diabetes (33.8%) were the most prevalent comorbidities in people hospitalized for COVID-19 [9]. In addition, patients with a history of hypertension, particularly in those without antihypertensive treatment or with poorly controlled status, are associated with higher risk of mortality for COVID-19 [10–12]. Thus, ensuring adherence to treatment to control blood pressure (BP) is challenging and may have prognosis impact in the context of the COVID-19 pandemic [12, 13].

Nonadherence to antihypertensive treatment is one of the main causes of inadequate BP control [14, 15] facilitating complications and target-organ damage, in addition to representing social and economic consequences [16–18]. The pandemic is requiring the adoption of restrictive measures for social distancing and isolation, which may influence the medical care routine (including the antihypertensive treatment) through barriers in accessing medication, reduced bonds with the health care network team or poor support network from family members and caregivers. Therefore, the objectives of the present study were to assess the adherence to antihypertensive drug treatment during the COVID-19 pandemic and to identify associated variables. We hypothesized that traditional factors related to poor adherence will continue to be relevant but the additional challenges for antihypertensive availability would have a role during the pandemic.

## Methods

### Study design and sampling

This is a cross-sectional study with consecutive hypertensive people attended at a tertiary hypertension outpatient clinic of a teaching hospital in the city of São Paulo, SP, Brazil. The outpatient hypertension service attends to about 800 hypertensive people that were referred by primary for specialized care. The nonprobabilistic sample was calculated based on an adherence rate of 50% according to a study carried out with the same population [19] and a significance level of 5%, resulting in 258 participants. However, all registered patients who met the inclusion criteria were considered for the study. Inclusion criteria comprised adults under pharmacological treatment for hypertension followed for at least 6 months. Beyond refuses to participate and not valid telephone number, we also excluded pregnancy and significant cognitive impairment.

### Measurements

The adherence to antihypertensive drug treatment was assessed using the 4-item Morisky Green Levine Scale [20]. The scale consists of questions that aim to assess adherence to treatment according to the participant's response being “Yes” or “No.” It is considered adherent to drug treatment when all responses are negative (final score, 0) and nonadherent when a score is ≥ 1. We did not use the 8-item Morisky Medication Adherence Scale because it is not free for using and require formal license and specific training. Moreover, recent evidence found that the performance of 4-item scale is comparable to the 8-item [21].

Information about COVID-19 and social distancing was assessed by asking questions during the telephone call including carrying out a previous or current positive diagnostic test for COVID-19 and the type of test (serology, nasopharyngeal swab or rapid reverse transcription polymerase chain reaction); medical care (in person) during the quarantine period related to BP control; leaving home and the frequency of leaving home; use of a protective mask; the number of people in the place of social isolation; isolation place in the home; contact with people diagnosed with COVID-19; difficulties in acquiring antihypertensive drugs during the pandemic; and occurrence of symptoms related to COVID-19 including fever, shortness of breath, loss of taste and/or smell, runny nose, headache, and body aches, at the time of the call or before, considering the period evaluated.

### Participant’s recruitment

Due to the obligation of social distancing during the period of the COVID-19 pandemic, data were collected by telephone, from March 24 to August 31, 2020. To identify eligible patients, the service's electronic medical record database was consulted. The interviews were carried out after at least three telephone contact attempts on different days and times. The free and informed consent form was read at the beginning of the interview and after the participant's verbal consent, data were collected. The research project was approved by the Research Ethics Committee (protocol nº 4088,764 and nº 4,093,932). Clinical and prescription variables for drug treatment were obtained from the electronic medical record. Uncontrolled BP was defined as BP ≥ 140/90 mmHg. Before measuring BP, it was made sure that the patient did not have a full bladder; did not exercise for at least 60 min; have not ingested alcoholic beverages, coffee, or food; and have not smoked in the last 30 min. The patients were kept seated: with their legs uncrossed, their feet flat on the floor, their backs on the chair and relaxed, and with the arm at the level of the heart and the palm facing upwards. A cuff suitable for the size of the arm was used. Three consecutive BP measurements were taken, with a validated automatic device, with an interval of 2 min between each measurement. The first BP value was excluded and the mean value of the last two measurements was used.

### Data analysis

The IBM SPSS ver. 25 (IBM Corp., Armonk, NY, USA) statistical software was used for data analysis. The significance level adopted was 5%. For categorical variables, Pearson chi-square test, Fisher exact test, or likelihood ratio were used, and for quantitative variables, Wilcoxon-Mann–Whitney, Brunner-Munzel, and Student t-test were used, depending on the variable distribution. For the multivariate analysis, a logistic regression model was used. The variables that entered the model were skin color (white, black and brown/yellow), age (mean), individual monthly income (mean), chronic kidney disease (yes or no), diabetes mellitus (yes or no), presence of symptoms related to COVID-19 (fever and body pain), currently works (yes or no) and having been without antihypertensive medication in some time of isolation (yes or no).

A subanalysis was also performed with previously collected data, where 111 participants in common were included. We used the McNemar test to compare adherence to antihypertensive pharmacological treatment before and during the COVID-19 pandemic.

## Results

**Fig. 1:**
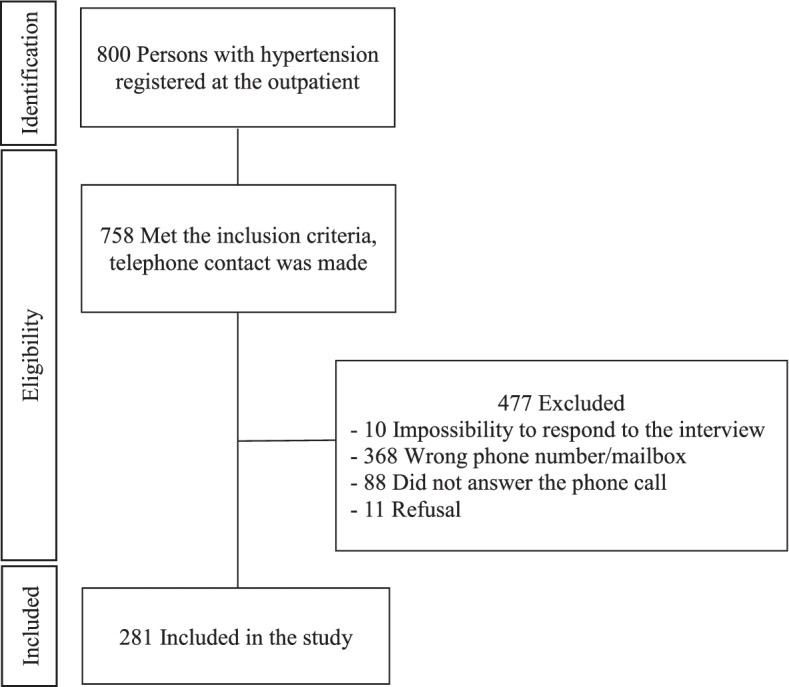
Flowchart of the inclusion and exclusion process of hypertensive patients during the COVID-19 pandemic

The flowchart of the process of inclusion and exclusion of participants is shown in Fig. 1. Approximately half of the interviewed participants (41.3%) were classified as nonadherence to antihypertensive drug treatment. The item that most contributed to nonadherence, according to the Morisky Green Levine Scale, was forgetfulness (35.6%) (Table 1).

**Table 1:** Adherence to antihypertensive drug treatment in hypertensive people during the COVID-19 pandemic (*n* = 281)

Adherence to antihypertensive drug treatment (Morisky Green Levine Scale)	Yes (%)	No (%)
Have you ever forgotten to take your medication?	100 (35.6)	181 (64.4)
Are you sometimes careless in taking your medication?	40 (14.2)	241 (85.8)
When you feel better, do you sometimes stop taking your medication?	8 (2.8)	273 (97.2)
Sometimes, if you feel worse when you take your medicine, do you stop taking it?	9 (3.2)	272 (96.8)

Table 2 presents the subanalysis of the 111 hypertensive patients comparing adherence to antihypertensive treatment before and during the pandemic. In this subsample, nonadherence was worse (*P* < 0.05) during the pandemic period, confirming the hypothesis of this manuscript.

**Table 2:** Comparison of adherence assessment before and during the COVID-19 pandemic (*n* = 111)

Adherence assessed	Adherence (%)		*P*-value
	Yes	No	
Before the pandemic period	99 (89.2)	12 (10.8)	0.000^a^
During the pandemic period	61 (54.9)	50 (45.1)	

^a^*P*-value obtained by McNemar test

The biosocial data in Table 3 show that most hypertensive patients were white females in the sixth decade of life. Approximately half of them were married and mean education of 9.0, standard deviation [SD], 4.0 years). The majority of hypertensive patients (75.8%) did not work, were retired (70.9%), and had a monthly income equivalent to US $325.7 (SD, US $238). Hypertensive patients who adhered to antihypertensive drug treatment in relation to nonadherent individuals presented a lower percentage of black people and older age (*P* ˂ 0.05 for all comparisons).

**Table 3:** Biosocial characteristics of adherents and nonadherent hypertensive people to antihypertensive drug treatment during the COVID-19 pandemic (*n* = 281)

Variable	Adherence			*P*-value
	Yes	No	Total	
Sex				0.575^a^
Female	105 (63.6)	70 (60.3)	175 (62.3)	
Male	60 (36.4)	46 (39.7)	106 (37.7)	
Skin color				0.001^b^
White	111 (67.3)	59 (50.9)	170 (60.5)	
Black	28 (17.0)	41 (35.3)	69 (24.6)	
Brown/yellow	26 (15.8)	16 (13.8)	42 (14.9)	
Marital status				0.309^a^
Married	75 (45.5)	57 (49.1)	132 (47.0)	
Unmarried	34 (20.6)	31 (26.7)	65 (23.1)	
Divorced	16 (9.7)	9 (7.8)	25 (8.9)	
Widow	40 (24.2)	19 (16.4)	59 (21.0)	
Age (yr)	67 ± 14	64 ± 14	66 ± 14	0.044^c^
Education (yr)	9.0 ± 4.2	9.1 ± 4.4	9.0 ± 4.0	0.613^a^
Currently work				
Yes	34 (20.6)	34 (29.3)	68 (24.2)	
No	131 (79.4)	82 (70.7)	213 (75.8)	0.093^a^
Individual monthly income (US$)	308.57 ± 247	350.39 ± 225	325.70 ± 238	0.149^c^
Family monthly income (US$)	527.98 ± 508	512.32 ± 288	521.53 ± 431	0.745^c^

Data are presented as number (%) or mean ± standard deviation

*P*-value obtained by ^a^Pearson chi-test,

^b^Fisher exact test, and

^c^t-test

About a third of hypertensive patients were former smokers and only 7.8% reported current smoking. The use of alcoholic beverages was reported by 19.9% and beer was the most consumed beverage (71.4%). The mean number of alcoholic beverages ingested on each occasion was three doses and just over a third of women and men consumed four and five doses, respectively. Almost all of the hypertensive patients (90.4%) were sedentary or became sedentary after the beginning of isolation due to the pandemic. The body mass index was at the upper end of the overweight range and the majority (80.1%) were overweight/obese. Dyslipidemia was the most prevalent morbidity, followed by diabetes and chronic kidney disease, and 15.3% had left ventricular hypertrophy.

About half of the individuals had uncontrolled BP. Adherent hypertensive individuals, compared with nonadherent hypertensives, had a longer history of coronary insufficiency and lower values of diastolic BP (*P* ˂ 0.05) (Table 4).

**Table 4:** Habits, lifestyle, comorbidities, and anthropometric data of adherents and nonadherent hypertensive people to antihypertensive drug treatment, during the COVID-19 pandemic (*n* = 281)

Variable	Adherence			*P*-value
	Yes	No	Total	
Smoking				
Yes	13 (7.9)	9 (7.8)	22 (7.8)	0.510^a^
No	99 (60.0)	77 (66.4)	176 (62.6)	
Ex-smoker	53 (32.1)	30 (25.9)	83 (29.5)	
Alcoholic beverage use				
Yes	32 (19.4)	24 (20.7)	56 (19.9)	0.543^a^
No	102 (61.8)	76 (65.5)	178 (63.3)	
Stopped	31 (18.8)	16 (13.8)	47 (16.7)	
Type of alcoholic beverage				
Beer	21 (65.6)	19 (79.2)	40 (71.4)	0.287^b^
Wine	8 (25.0)	2 (8.3)	10 (17.9)	
Distillates (whiskey, brandy, vodka, liquor, cachaça)	3 (9.4)	3 (12.5)	6 (10.7)	
Amount of alcoholic beverage intake (dose)	3.0 ± 3.0	4.0 ± 2.0	3.0 ± 3.0	0.143^c^
Intake ≥ 5 servings for men and ≥ 4 servings for women	10 (31.3)	9 (37.5)	19 (33.9)	0.625^a^
Physical activity				
Sedentary	95 (57.6)	70 (60.3)	165 (58.7)	0.433^a^
Stopped after the start of the pandemic	51 (30.9)	38 (32.8)	89 (31.7)	
Active	19 (11.5)	8 (6.9)	27 (9.6)	
Body mass index (kg/m^2^)	29.6 ± 5.6	29.8 ± 5.4	29.7 ± 5.5	0.732^c^
Nutritional status				
Eutrophic	33 (20.5)	22 (19.1)	55 (19.9)	0.581^a^
Overweight	61 (37.9)	38 (33.0)	99 (35.9)	
Obesity	67 (41.6)	55 (47.8)	122 (44.2)	
Personal background				
Secondary hypertension	12 (7.3)	8 (6.9)	20 (7.1)	0.904^a^
Resistant hypertension	42 (25.5)	31 (26.7)	73 (26.0)	0.811^a^
Coronary insufficiency	17 (10.3)	4 (3.4)	21 (7.6)	0.031^a^
Dyslipidemia	92 (55.8)	61 (52.6)	153 (54.4)	0.599^a^
Diabetes mellitus	77 (46.7)	42 (36.2)	119 (42.3)	0.081^a^
Chronic kidney disease	54 (32.7)	28 (24.1)	82 (29.2)	0.119^a^
Stroke	15 (9.1)	12 (10.3)	27 (9.6)	0.725^a^
Left ventricular hypertrophy	29 (17.6)	14 (12.1)	43 (15.3)	0.207^a^
SBP (mmHg)	139.2 ± 22.1	137.6 ± 23.7	138.5 ± 22.7	0.558^c^
DBP (mmHg)	75.8 ± 14.1	79.4 ± 14.6	77.3 ± 14.4	0.040^c^
Control (SBP < 140 and DBP < 90)				
Yes	81 (50.3)	61 (53.5)	142 (51.6)	0.601^c^
No	80 (49.7)	53 (46.5)	133 (48.4)	

Data are presented as number (%) or mean ± standard deviation

*SBP* Systolic blood pressure, *DBP* Diastolic blood pressure

*P*-value obtained by ^a^Pearson chi-test,

^b^Fisher exact test, and

^c^t-test

The total mean ± standard deviation of medications prescribed by hypertensive patients was 7.4 (SD, 2.8), 3.2 (SD, 1.2) were antihypertensive and of these, 42% used four or more different classes of antihypertensives. Just over half of the hypertensive patients (55.2%) received their medication at home by mail. However, the majority (63.9%) reported problems in receiving medications during the pandemic, due to delays, nondelivery, or incomplete delivery. There were also difficulties in obtaining antihypertensive drugs in the primary healthcare network, such as the lack of medication and a medical prescription with an expired date. Nonadherent hypertensive individuals, compared with adherent hypertensives, reported having been longer without an antihypertensive during the pandemic period evaluated (*P* ˂ 0.05) (Table 5).

**Table 5:** Drug treatment of adherents and nonadherent hypertensive people drug treatment, during the COVID-19 pandemic

Variable	Adherence			*P*-value
	Yes	No	Total	
No. of prescribed drugs	7.1 ± 2.6	6.8 ± 2.6	7.4 ± 2.8	0.412^a^
No. of antihypertensives prescribed	3.2 ± 1.1	3.3 ± 1.2	3.2 ± 1.2	0.563^b^
1	10 (6.1)	8 (6.9)	18 (6.0)	0.645^a^
2–3	89 (53.9)	56 (48.3)	145 (52.0)	
≥ 4	66 (40.0)	52 (44.8)	118 (42.0)	
Acquisition of antihypertensive drugs				
Buy	11 (6.7)	8 (6.9)	19 (6.8)	0.203^c^
Pick it up directly at the hospital pharmacy or popular pharmacy	27 (16.4)	25 (21.6)	52 (18.5)	
Receive from the hospital at home by mail	88 (53.3)	67 (57.7)	155 (55.2)	
Pick up at the health center	39 (23.6)	16 (13.8)	55 (19.5)	
Difficulties during the pandemic to acquire antihypertensive drugs				
Hospital related				
Failed to deliver or was delayed	33 (37.5)	31 (46.3)	64 (22.6)	0.396^c^
Partially received the medicines	23 (26.1)	12 (17.9)	35 (41.3)	
There was no difficulty	32 (36.4)	24 (35.8)	56 (36.1)	
Health center related				
Yes	11 (28.2)	6 (37.5)	17 (30.9)	0.533^c^
No	28 (71.8)	10 (62.5)	38 (69.1)	
Ran out of antihypertensive medication during quarantine				
Yes	10 (6.1)	18 (15.5)	28 (10.0)	0.009^b^
No	155 (93.9)	98 (84.5)	253 (90.0)	

Data are presented as mean ± standard deviation or number (%)

*P*-value obtained by ^a^t-test,

^b^likelihood ratio, and

^c^Pearson chi-test

Almost half of the hypertensive patients (49.8%) had their medical appointments at the hypertension service suspended due to the pandemic, and for the others, the appointment was maintained due to unsuccessful telephone contact or the patient’s severity. Among those who had their appointment suspended, the majority (69.3%) was rescheduled for a maximum period of 4 months after the cancellation date. Part of the patients (24.9%) needed some health care during the period, 11 people reported that it was related to COVID-19 symptoms (nine patients had a confirmed diagnosis of COVID-19 and five required hospitalization). The majority (77.6%) reported leaving their home during the COVID-19 isolation period. For those who left home more than 12 times (38.1%), the main reason was to resume to work. Almost all participants reported using a protective mask on the occasions when they left home. Hypertensive patients also reported feeling insecure about leaving the house, even after the relaxation of isolation measures (Table 6).

**Table 6:** Health conditions of adherents and nonadherent hypertensive people to antihypertensive drug treatment, during the COVID-19 pandemic (*n* = 281)

Variable	Adherence			*P*-value
	Yes	No	Total	
Rescheduling of medical appointment				
Yes	84 (50.9)	56 (48.3)	140 (49.8)	0.667^a^
No	81 (49.1)	60 (51.7)	141 (50.2)	
Medical appointment rescheduling time (mo)				
1–4	56 (68.3)	39 (70.9)	95 (69.3)	0.954^b^
4–6	21 (25.6)	13 (23.6)	34 (24.8)	
≥ 7	5 (6.1)	3 (5.5)	8 (5.9)	
Sought after health care during the pandemic period				
Yes	42 (25.5)	28 (24.1)	82 (24.9)	0.802^a^
No	123 (74.5)	88 (75.9)	211 (75.1)	
Reasons for medical care				
Symptoms related to COVID-19	7 (16.7)	4 (14.3)	11 (15.7)	1.000^b^
Routine or emergency consultation	35 (83.3)	24 (85.7)	59 (84.3)	
Sought after medical care during the pandemic period due to hypertension				
Yes	5 (3.0)	7 (6.0)	12 (4.3)	0.243^c^
No	160 (97.0)	109 (94.0)	269 (95.7)	
COVID-19 medical diagnosis				
Yes	6 (4.0)	3 (2.6)	9 (3.2)	0.623^a^
No	159 (96.0)	113 (97.4)	272 (96.8)	
Left home during quarantine (time)				
Yes	124 (56.9)	94 (43.1)	218 (77.6)	0.244^a^
1–4	55 (44.7)	31 (33.0)	86 (39.4)	0.042^c^
5–8	20 (16.3)	12 (12.8)	32 (14.7)	
9–12	11 (8.9)	5 (5.3)	16 (7.3)	
> 12	37 (30.1)	46 (48.9)	83 (38.6)	
Wore a mask when leaving home				
Yes	123 (99.2)	92 (97.9)	215 (98.6)	0.408^b^
No/sometimes	1 (0.8)	2 (2.1)	3 (1.4)	
Contact with people with COVID-19				
Yes	24 (14.7)	14 (12.0)	38 (13.5)	0.667^a^
No	139 (85.3)	102 (88.0)	243 (86.5)	
Felt safe to leave the house after decreasing restriction measures				
Yes	11 (6.7)	12 (10.3)	23 (8.2)	0.268^b^
No	154 (93.3)	104 (89.7)	258 (91.8)	

*P*-value obtained by ^a^Pearson chi-test,

^b^Fisher exact test, and

^c^t-test

The multiple regression model (Table 7) showed that (P < 0.05) among black hypertensive individuals, the chance of nonadherence was 2.6 times greater when compared to whites. Among those who were without antihypertensive medication at some time during isolation, the chance of nonadherence increased by 2.5 times.

**Table 7:** Variables associated with nonadherence to antihypertensive drug treatment, during the COVID-19 pandemic

Variable	Adjusted OR (CI 95%)	*P*-value
Skin color		
White	1	
Black	2.620 (1.464–4.688)	0.001
Brown/yellow	1.173 (0.580–2.373)	0.656
Having been without antihypertensive medication in some time of isolation		
No	1	
Yes	2.562 (1.114–5.891)	0.026

Variables included in multiple regression: skin color, age, individual monthly income, chronic kidney disease, diabetes mellitus, presence of symptoms related to COVID-19, currently works, and having been without antihypertensive medication in some time of isolation

*OR* Odds ratio, *CI* Confidence interval

## Discussion

The present study analyzed the factors associated with nonadherence to antihypertensive treatment in hypertensive patients treated at a specialized outpatient unit during the pandemic. Black skin color and having been without antihypertensive medication in some time during isolation were variables that were independently associated with nonadherence.

The prevalence of nonadherence to antihypertensive treatment observed in our study (41.3%) was similar or even lower than reported in the literature [22–24] but much higher than observed in another study (17.8%) carried out with the same population [25]. These studies, however, were carried out outside the context of the COVID-19 pandemic. Therefore, isolation due to the COVID-19 pandemic may have contributed to reduced adherence to treatment, as verified in the sample subanalysis. Despite the fact that most hypertensive patients reported were adherent to antihypertensive treatment, a very high number was found to be nonadherent. Forgetfulness was the main reason that contributed to the lack of adherence and can be explained by the complexity of the treatment, polypharmacy, and cognitive alterations [26–28].

It is also noteworthy that a large part of the participants had other comorbidities and/or complications related to hypertension and adherence to the treatment of these diseases may also have been impaired. Thus, in the context of the COVID-19 pandemic, it is important to consider the possibility of a worsening of the health condition, which could result in important complications, such as target-organ damage. Recent guidelines reinforce the urgent need to consider adherence as a fundamental issue for achieving success in the treatment of hypertension [16, 17, 29–32].

The assessment of biosocial characteristics showed that the black skin color was predominant in nonadherent hypertensive patients. Black hypertensive individuals had a greater chance of nonadherence to antihypertensive treatment when compared to whites. In the context of the disease and treatment of hypertension, the influence of the black skin color, when compared to whites, has been previously described in the literature [33–37]. The higher prevalence of unfavorable socioeconomic conditions, less access to health services and inadequate lifestyle habits observed in Blacks can directly influence the adequate adherence to treatment. Regarding antihypertensive drug therapy, most of the sample used combinations of two to three or four or more drug classes. This high number of antihypertensive drugs is possibly related to the severity profile of hypertensive patients. Intermittent access to antihypertensive medication at home someday during the isolation period increased the chance of nonadherence by 2.3 times, as access to medications is an essential condition for satisfactory adherence to antihypertensive treatment [16, 27]. In Brazil, the distribution of medicines is carried out free of charge by the Unified Public Health System. For that, a valid medical prescription is needed. In the present study, most hypertensive patients received their medication free of charge at home, as a routine adopted by the institution where the study was conducted, but this flow was impaired during the pandemic period. This was also observed in another study in which it was observed difficulty in accessing medicines during the pandemic in 35% of the participants and the main reasons were limited availability of means of transport, reduced monthly income, increase in the value of medicines, in addition to the fear of leaving home and being infected by the virus [38]. The pandemic changed this routine with failure to deliver medication during the isolation period. The adaptation of flows to the pandemic context would possibly allow access to continuous-use medicines to a greater number of people. Adherence to treatment is a complex process in which several factors interact. The problem of accessibility to medicines and the consequent worsening of adherence has already been highlighted in the literature, which emphasizes the dimension defined by the World Health Organization, in which social and economic conditions are decisive in the adherence process. There is also the influence of knowledge about the disease, such as chronicity and health beliefs; in relation to the treatment, the treatment for life, undesirable effects, and costs are highlighted; irregular follow-up to health services; and dissatisfaction with the treatment also interfere with treatment adherence [27, 39–41].

It is noteworthy that during the study period, few hypertensive patients reported having a confirmed diagnosis of COVID-19, requiring hospitalization. Hypertension can be considered a high-risk factor for an unfavorable prognosis in COVID-19 [6, 42, 43]. The low prevalence of COVID-19 in the studied sample may be due to the high adherence to social isolation measures, and when it was inevitable to leave the house, they adopted individual protection measures with the use of a mask. However, it is important to highlight that not all those studied underwent a test for COVID-19 and may have had the disease asymptomatically.

This study has strengths and limitations. It was possible to indirectly measure nonadherence to drug treatment for hypertension in the population of hypertensive patients monitored at the hypertension outpatient clinic of the largest public hospital complex in Latin America and to know the factors associated with nonadherence to antihypertensive treatment during the context of the COVID-19 pandemic allows for the establishment of targeted measures and optimization of scarce resources for greater effectiveness. The limitations of the present study are related to the study design (cross-sectional) does not allow the establishment of the cause-and-effect relationship. In addition, the data obtained represent the population of a specialized hospital and cannot be generalized. And the data obtained through telephone contact, being subject to the effects of social desirability and memory bias, in addition to the impossibility of contacting everyone patients registered at the outpatient, which may have caused a selection bias.

## Conclusions

This is first study to evaluate adherence to drug antihypertensive treatment during the COVID-19 pandemic. The prevalence of nonadherence to antihypertensive drug treatment was high, and sociodemographic variables, as well as the lack of antihypertensive medication were associated with nonadherence to treatment. The COVID-19 pandemic imposed substantial social restriction impairing the medical care routine, which may have influenced adherence to the antihypertensive treatment.

## Data Availability

The data sets generated during and/or analyzed during the current study are available from the corresponding authors on reasonable request.

## Abbreviations

BP: Blood pressure
CI: Confidence interval
COVID-19: Coronavirus disease
DBP: Diastolic blood pressure
OR: Odds ratio
SBP: Systolic blood pressure
SD: Standard deviation
